# Analyzing drivers of organic food sales–A pooled spatial data analysis for Hamburg (Germany)

**DOI:** 10.1371/journal.pone.0285377

**Published:** 2023-10-04

**Authors:** Sarah Joseph, Hanno Friedrich

**Affiliations:** Kühne Logistics University (KLU), Hamburg, Germany; Sichuan Agricultural University, CHINA

## Abstract

Shifting the food system to a more sustainable one requires changes on both sides of the supply chain, with the consumer playing a key role. Therefore, understanding the factors that positively correlate with increased organic food sales over time for an entire population can help guide policymakers, industry, and research to increase this transition further. Using a statistical approach, we developed a spatial pooled cross-sectional model to analyze factors that positively correlate with an increased demand for organic food sales over 20 years (1999–2019) for an entire region (the city-state of Hamburg, Germany), accounting for spatial effects through the spatial error model, spatially lagged X model, and spatial Durbin error model. The results indicated that voting behavior strongly correlated with increased organic food sales over time. Specifically, areas with a higher number of residents that voted for a political party with a core focus on environmental issues, the Greens and the Left Party in Germany. However, there is a stronger connection with the more “radical” Left Party than with the “mainstream” Green Party, which may provide evidence for the attitude-behavior gap, as Left Party supporters are very convinced of their attitudes (pro-environment) and behavior thus follows. By including time and space, this analysis is the first to summarize developments over time for a metropolitan population while accounting for spatial effects and identifying areas for targeted marketing that need further motivation to increase organic food sales.

## Introduction

At the individual level, buying behavior is an important instrument for consumers to express their particular attitudes and take action, especially for regular purchases such as food. What we eat and how it is produced significantly impacts environmental sustainability today as well as climate change consequences and food security in the future [1]. The potential to produce sufficient food to feed the ever-increasing population depends on our ability to shift the food system to one that enhances resource efficiency and reduces environmental impacts through sustainable consumption patterns and production methods. Improvements are necessary across the food supply chain, and consumer demand plays a key role [2, 3].

Industrialized agriculture, characterized by the heavy use of monocultures, high inputs of chemical and mineral fertilizers, and large-scale meat and dairy production based on concentrated animal feeding operations (CAFOs), is responsible for producing most of the world’s food [4] However, according to the Food and Agriculture Organization of the United Nations (FAO), systems are at a “breaking point,” and current patterns of industrialized agriculture are not sustainable; productive capacity is being pushed to the limit, and land and ecosystems are severely degraded [5].

Several studies have examined the potential for more sustainable production methods that focus on maintaining the health of soils and ecosystems and meeting the demand for food, such as sustainable intensification [6, 7], regenerative agriculture [8], and, most notably, organic agriculture [9–18]. Organic agriculture is a broad term used to describe a production system to enhance the health of soils, ecosystems, and people by restricting synthetic pesticides, herbicides, and fertilizers. Rather, relying instead on ecological processes, biodiversity, and natural cycles adapted to local conditions [19]. Furthermore, organic certification (visible through a label on a product or packaging) ensures that the product is produced according to specific standards, which vary from the most basic national or regional certification (such as the European Union or German) to more ambitious farming associations.

Although the conversion to organic agriculture may require more resources (such as land, farmer knowledge, or manual labor), the production method aims to sustain or enhance the health of soils and the environment from a long-term perspective [5, 20]. Furthermore, compared to other economic sectors, agriculture is disproportionally at risk of climate change-related hazards such as extreme weather events (short-term) and slow-onset stressors (long-term), including increasing temperatures, land degradation, and biodiversity loss. Over time, these stressors can negatively affect the ability of an ecosystem to cope with changes (such as increasing droughts or flooding) [21]. Thus, the role of food production must be considered in terms of long-term impacts, and organic agriculture (or other ecological production methods) can potentially reduce the environmental impacts of our current industrialized food system.

Although many [5, 22, 23] have called for a shift to more sustainable production methods, the market share for organic food products remains low. Compared with the total food market, the share of organic food sales is still small, approximately 6.4% in Germany [24] and 2.8% globally [25, 26] in 2020. Access to affordable organic and sustainably produced foods is a major limitation in lower-income countries and globally [1]. What is considered “affordable” is relative to each individual and/or household income, and thus, the consumer’s attitude and willingness-to-pay (WTP) and the price premium associated with organic products play a considerable role. Currently, the market is not properly controlled; the “hidden costs” and environmental externalities associated with the industrialized food system, such as damage to soil or ecosystems, are often not reflected in the cost the consumer pays [1]. A study by the German government-initiated *Commission for the Future of Agriculture* (ZKL) found that the domestic farming sector caused externalities of approximately 90 billion euros annually [27]. In addition, many subsidies for agriculture are unrelated to climate or environmental performance; thus, prices for industrialized food products typically do not reflect the true cost of production (i.e., including externalities) [28].

In contrast, sustainable food production tends to reduce externalized costs instead of passing the burden on to society, but this includes a price premium paid by the consumer. Until the price of the product paid by the consumer reflects the true cost, the consumers’ WTP for sustainable food products is necessary for growth. In Germany (the second largest organic food market globally after the U.S.), the market grew from approximately two to twelve billion euros between 1999 and 2019 [29]. Transparency in the food supply chain is becoming increasingly important, and consumers are increasingly interested in knowing how their food is produced and the impact of that production on the environment and society, thus resulting in a turn to more ecological products [1, 24]. As observed in higher-income regions, such as North America and Europe, an increase in consumer demand has also increased the production and certification of ecological products, consequently significantly reducing their prices [1]. Thus, consumer demand continues to be the driving force behind the increase in sustainably produced food products.

The question arises: What part of the population currently buys organic and/or ecological food products? To understand the development of the drive in demand for organic food products, examining organic food sales over time within larger populations is necessary. Although the organic market has shifted from a very small percentage of the population to larger groups, it remains a niche, and some areas exhibit no demand for organic and/or ecologically produced products. Thus, the growth potential of organic food sales may be limited if the factors that drive growth over time to new target markets and the conditions that can foster an increase in organic food sales for an entire population are not addressed. This includes a focus on those demographics that may be difficult to reach with surveys of smaller groups at single points in time, as has often been done in research thus far.

Therefore, this study aimed to identify groups that positively correlate with increased organic food sales using a spatially pooled cross-sectional model. Data were collected over 20 years (1999–2019) for the entire population (approximately 1.7 million inhabitants in 1999 to 1.9 million in 2019) of a large metropolitan city (Hamburg, Germany). We analyzed the relationship between organic food sales from all (to the best of our knowledge) food retailers in Hamburg during the entire period and the surrounding environment (inhabitants and infrastructure), taking into account potential spatial effects (i.e., the influence of neighboring observations, not accounted for in previous studies).

Several studies have also examined the drivers of sustainable food consumption, especially in relation to sociodemographic indicators, individual attitudes, and accessibility. Generally, higher education levels consistently positively correlate with sustainable food sales, but the relationships among other indicators such as age, gender, income, and accessibility vary. Recent studies suggest that individual attitudes towards sustainability tend to correlate more strongly with sustainable food sales than sociodemographic factors or that sociodemographic aspects play a moderating role in shaping attitudes towards organic food sales [30–36]. To date, most studies have collected data in two ways: survey data or true purchase data combined with surveys. Survey data collection included stated preferences [30–36], revealed preferences [37, 38], or both [39]. Studies using true purchase data [40–43] obtain this from nationwide panels or from specific retailers that track the household purchase behavior of participants for a specific time period, such as over one year or less [40, 41, 42] or a two-year period [43], and combine this with self-reported behavior on purchase intention and/or attitudes towards organic food or sustainability.

However, as indicated by Moser (2015) [44] and acknowledged by others [42, 43, 45–47] self-reported behavior is not always transferred to true purchasing behavior (i.e., the attitude-behavior gap). Janssen (2018) reconfirmed Moser’s (2015) finding that data on actual purchasing behavior is a better basis for further research and has the potential to provide more accurate results. Therefore, many studies have combined true purchase data on specific organic products [48–52] or organic food in general [40–43] to generate findings. However, these studies still rely on consumer surveys or self-reporting to indicate participants’ attitudes toward sustainability and/or organic foods. Thus, consumers may still overestimate their preferences or attitudes towards sustainability, ecological products, and/or organic food, even when combined with actual purchase data, owing to biases such as social desirability (responding in a way that will be viewed favorably by others) or acquiescence (responding in a way that agrees with research statements or the interviewer). This further exemplifies the need for studies that include data describing consumers that are not collected via surveys.

To fill this research gap, we investigated drivers of the demand for organic products over time for an entire population from the sales perspective rather than the individual consumer (i.e., no self-reported data). As it was not feasible to collect individual store data for each year over the time period (because the dataset included more than 4,500 observations of retailers spread across 20 years and multiple chain and single-owner shops), we reasonably inferred the organic sales of each individual store for each time period using secondary data from company or industry reports, as well as data purchased from *Trade Dimensions* (a commercial data provider) collected from the retailers. In addition, data on residents were collected via the Northern German statistical database *Statistik Nord* (the local statistical office) and included information on sociodemographic characteristics and voting behavior. This is discussed further in section three. By pooling data over a two-decade period, we identified robust drivers in demand for organic food (which may help predict future trends) over a long period and could avoid a potential bias of data collected at a single point in time or over just a few years.

Furthermore, we estimated the model using standard linear modeling (SLM) to address the entire population of a region over an extended period, as well as developed a spatial pooled cross-sectional model to account for the effect of unobserved spatial effects (e.g., spatial dependence) of neighboring areas on the dependent variable, explanatory variables, and the error term that had not yet been considered in the current literature. In addition to being a challenge, however, this spatial aspect also creates value for potential stakeholders, such as policymakers or industry, to illustrate on the map where organic sales thrive or to identify target areas (or neighborhoods) that may require more attention or motivation to promote sustainable food purchases.

By considering the macro perspective for both sales and characteristics of residents and infrastructure, we removed bias from respondents (social desirability and/or acquiescence), as well as selection bias in the sample. Unlike previous studies, the data in this study was not self-reported by the consumer for purchase behavior, characteristics, or attitudes. Thus, the results did not group consumers by their personally stated opinions on environmental aspects or sustainability; rather, we identified groups that positively correlate with organic food sales over time for an entire region based on the data itself. To the best of our knowledge, this is the first study to examine the determinants of organic food purchases using a macro approach to remove bias. Additionally, this is the first study to analyze data over a 20-year period, as the next longest studies that include participants of all ages [37, 53] are from a four period. Finally, this is one of only two studies considering spatial effects, which may have implications for both results and practical relevance. Unlike Li et al. (2018), who analyzed the location of organic retailers, this study also considers organic food sales from conventional retailers.

The rest of the study is organized as follows. An overview of drivers for organic food consumption is presented in the next section, and the subsequent four sections describe the data, model specification, estimation of results, and empirical findings. Finally, the discussion and conclusions section follows.

## Literature review: Drivers of organic food consumption

Many studies have examined the determinants of organic food consumption, especially in terms of specific sociodemographic characteristics or attitudes as well as accessibility. However, as described above, data collection is typically performed via surveys or surveys combined with true purchase data, which may lead to bias in the results. To clearly illustrate the research gap filled by this study, we first divided the literature review by the main drivers identified in previous studies (sociodemographics, attitudes, and accessibility). We also identified the methodology used (survey data, true purchase data, or both) and significant findings within each section. Finally, we also introduced the literature that focused on voting behavior as an indicator and/or driver of environmental attitudes to provide the theoretical background for the main findings of this study.

### Sociodemographic factors and preference for organic food

Early studies associating consumers’ sociodemographic characteristics with demand for organic products emerged as early as the 1980s [54]. Since then, several studies have been conducted, although the results have not been consistent across studies, regardless of the data collection method. For example, some studies that used only survey data found a positive association between organic consumption and being older [31, 38], being female [31, 38], having higher education levels [30, 35, 37], and earning higher income [38]. However, other studies have found no significant differences in terms of income [30], age [30] or family size [30, 31]. Studies that have combined true purchase data with survey data have also reported contradictory findings. For example, some found a positive correlation between organic food purchases and being female [40], earning higher income [42, 48, 51], having higher education [40, 42, 48, 50, 51], or having young children in the home [42], whereas others found a negative influence from being older [42, 50] or from having more children in the home [51]. Generally, studies have consistently found that education positively correlates with organic food sales, while the influence of other sociodemographic characteristics such as age, gender, and income varies.

### Attitudes toward sustainability and organic food

Recent research, however, suggest that individual attitudes toward sustainability tend to be more important than sociodemographic factors or that sociodemographic factors play a moderating role in shaping attitudes toward organic food purchases and behavior [31]. Therefore, studies typically report results in two ways: (1) identifying motivations that shape their attitudes towards organic food (e.g., concern for animal welfare); or (2) clustering participants based on either self-reported behavior (e.g., frequent, occasional, or non-consumers) and then identifying the characteristics of the various groups (which may also include sociodemographic factors).

Generally, research has consistently reported a significant correlation between a positive attitude towards sustainability and environmental protection with increased preference for organic food/organic food purchases across studies using only survey data as well as those that include true purchase data. Padilla Bravo et al. (2013) for example, examined data from the German National Nutrition Survey (n = 13,074), indicating that altruistic motives such as concern for animal welfare or the environment significantly impact purchase behavior, making sociodemographic variables less important. Similarly, Janssen (2018) combined true purchase data with survey data and found that concepts such as “naturalness,” “healthiness,” and “environmental protection” play a significant role in positive attitudes toward organic products. Several other studies have repeated these findings [36, 39, 41, 55–58].

Studies that cluster consumers based on self-reported behavior have also shown similar results. Kesse-Guyot et al. (2013) found that regular and occasional organic food consumers perceived organic food products as healthier and better for the environment. Similarly, Nasir and Karakaya (2014) found that those with a favorable attitude towards organic food exhibited higher health orientation and socially responsible consumption behavior. Ditlevsen et al. (2020) found that organic food consumers were more likely than non-consumers to include environmental factors in their decision-making process.

However, the attitude-behavior gap becomes apparent when true purchase data is combined with surveys on stated preferences towards organic food, as illustrated in Moser (2015). The author finds that despite a customer’s stated preference towards “greener” products, WTP was the strongest predictor of green purchasing behavior, and individuals overestimate their self-reported WTP and behavior [44]. This was acknowledged by Grimmer and Miles (2017), who found that the intention to purchase ecological products plays a role in predicting behavior, but this is mediated by the presence of a plan to purchase (e.g., to buy a specific eco-friendly product on their next shopping trip) and that the plan itself is formed by existence (has a plan been made or not?), and strength (degree of commitment) [59]. Thus, being convinced to purchase an ecological product is the strongest indicator of buying behavior.

### Accessibility to organic food

Several studies have also focused on accessibility to fresh and healthy foods regarding nutrition [60, 61] or shopping behavior [62, 63], with many including spatial data in their analysis [64–68]. Some studies have also accounted for spatial effects on food consumption or accessibility using spatial econometric models [69–72]. To the best of our knowledge, only one other study [53] has analyzed the accessibility of organic food with neighborhood or resident characteristics and found that the location of organic food retailers was positively associated with population density, education, and median housing value, with no disparities based on income. However, the authors did not consider organic food sales but rather the location of the retailers.

### Voting behavior, attitudes, and ecological preferences

Several studies dating back to the 1970s have also examined the role of ideologies such as political values in predicting environmental attitudes or sustainable behavior, generally finding that “green” voters tend to vote for “green” parties [73]. For example, Dunlap (1975) conducted a survey on political alignment and attitudes toward the environment among university students and found that those on the liberal left exhibited higher rates of pro-environmentalism than conservative students [74]. This sentiment was revealed by further research, although the vast majority of datasets were from the USA [75–81] or Asia [82, 83]. Nawrotzki (2012) explored this phenomenon (that liberals are generally more environmentally concerned than conservatives) from a more global perspective spanning 19 countries (including Germany). The study showed that conservatives’ support for environmental issues varies by country; conservatives in developed, capitalist nations with better environmental conditions are less concerned than liberals in those countries, whereas those in less developed countries that are characterized by poorer environmental conditions are more concerned than liberals in those countries [84]. This is also aligned with the findings of Papp (2022), who used a dataset covering 139 surveys across 38 countries between 1995 and 2016 and indicated that exposure to country-level environmental problems can increase the “green” vote cast by citizens, even if they are not particularly concerned about the environment [73]. This may imply that increased knowledge (e.g., observing the impacts of climate change or environmental problems firsthand) can also increase support for sustainability initiatives.

In addition, Ziegler (2017) analyzed the role of political orientation on climate change beliefs and WTP as a price premium for climate-friendly items in the USA, Germany, and China. The study found that while political orientation (liberal vs. conservative) was highly relevant to belief in climate change in the USA, it is not as important in Germany or China, where belief in climate change is firmer across all ideologies [85]. The study did find, however, that conservatives in the USA and Germany who did not align themselves with “green” politics were less likely to pay the price premium associated with climate-friendly products; therefore, a connection with “green” politics plays a role in WTP and purchase intention for ecological products [85]. In this case, “green” politics refers to a platform or political support for climate-friendly measures or initiatives, such as sustainably produced food.

Furthermore, research grounded in social identity theory (SIT) also posit that groups can shape personal attitudes and beliefs that one is a part of, such as social categories (e.g., gender or ethnicity), interest-based groups (e.g., environmental groups), professions (e.g., professional associations) [86], and political parties (e.g., Democrat or Republican in the U.S.) [87]. This also extends to the context of attitudes towards sustainability, implying that membership in specific groups can foster a positive (or negative) attitude towards pro-environmental behavior, such as a preference for organic food, depending on the attitudes of the group [86, 88, 89]. Thus, voting behavior can be a predictor of environmental attitudes, and alignment with a specific political party can further shape attitudes and behavior. However, this could be influenced by other factors, such as exposure to environmental problems [73] or the individual’s WTP for ecological products [85]. Furthermore, the attitude-behavior gap indicates that a preference for ecological products may not translate into buying behavior. Therefore, in this study, we aim to examine the relationship between political behavior and organic food purchases as well as analyze potential differences in WTP among political parties, which has not yet been addressed in the literature.

## Data

### Explained (dependent) variable: Organic food sales

We analyzed organic food sales from individual grocery retailers (N = 4934) in Hamburg, Germany, spanning 1999–2019. Organic food sales were selected as the variable to be explained because organic certification (assumed to be base-level EU Bio or German “*Bio-Siegel*”) implies increased transparency and ecological friendliness compared to conventionally produced food.

We used a bottom-up approach to calculate the organic food sales of each shop. First, data on the locations of all full-service, discount, and organic food retailers in Hamburg over the 20 years of 1999–2019, for every two years (i.e., 1999, 2001, 2003, …, 2019) were purchased from *Trade Dimensions*, which were directly collected from the retailers. Included in these data were the address of retailers (street name, street number, zip code, city, country), size of retailers (in square meters), name of the retailer (which may be the name of the retail chain), and warehouses. As a large portion of organic food sales came from traditional outlets and not just organic food shops [90], we also included these retailers in the analysis and applied an estimation of organic food sales as a portion of their total share of food sales (to be discussed in the following section).

Next, we collected data on average sales per square meter in terms of total food sales (for full-service retailers and discounters) and organic food sales (organic food retailers). As different chains generate different sales levels per square meter, we broke this value down by chain; for example, the full-service retailer *Rewe* has a different sales value per square meter than the full-service retailer *Edeka*, which is also different from the discounter *Lidl*. Sales per square meter by chain were also broken down by year for 1999–2019. In the case where there were no sales per square meter reported for a specific shop (either no company report was available or it was a single-owner shop), we used the average of all retailers broken down by type (i.e., discounter was the average of all discounters) for that year. The sales per square meter of the organic retail chains were purchased from Trade Dimensions, which provides the average of all shops per chain in Germany for that given year. For single-owner organic shops (referred to as “natural food stores” in Germany), in which the sales per square meter were not reported, we took the average of all organic food retailers and reduced this value by 10% according to the report from the German Federal Office for Food and Agriculture (BÖLN) [91].

**Fig 1:**
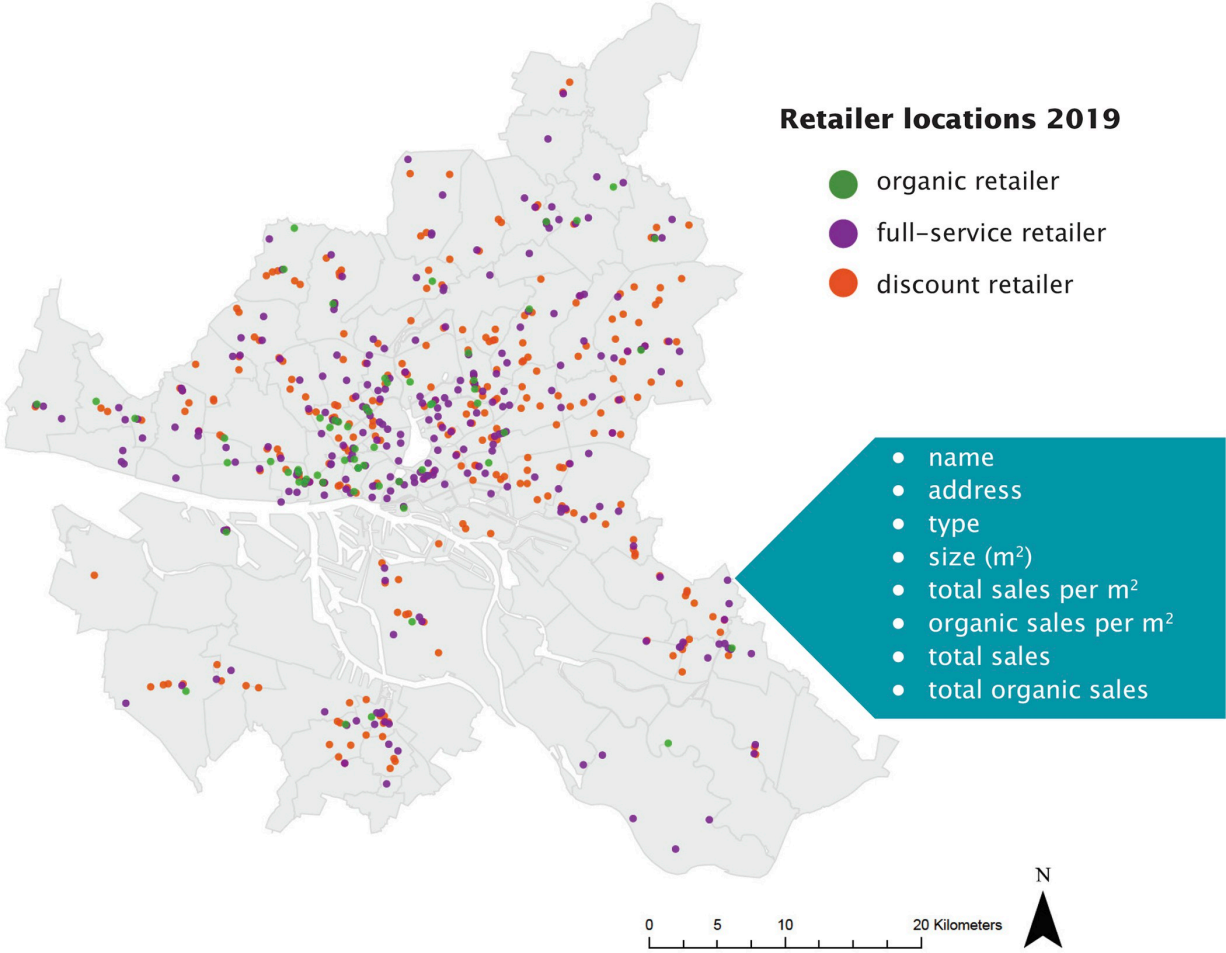
Retailer locations in 2019 (Hamburg, DE), broken down by type and including data description. Source: Maps generated with own data in ArcGIS Pro.

A further step was required for full-service and discount retailers to calculate the organic sales per square meter for each shop. Sales per square meter from organic shops were assumed to be 100% organic because all food products sold there were certified organic. For the other retailers, we collected data on the proportion of organic food sales in their total assortment, broken down by retailer type/chain and year. Data were collected from industry and company reports and used to calculate the average percentage of sales from the shop type (full-service or discounter), which was organic food sales for each year. This was then multiplied by the total sales per square meter for each shop. Following this step, we estimated the value for organic sales per square meter, broken down by shop and year. Finally, we multiplied the value of organic sales per square meter of each shop by the size of the shop to estimate the value of total organic sales from each shop per year. Fig 1 shows an example of retailer locations in 2019.

**Putting this into formal notation:**

$$O_{it}=S_{it}*M_{it}$$ (1)

$$M_{it}=T_{it}*p_{it}$$ (2)

where *O*_*it*_ is the total organic food sales from food retailer *i* at time period *t*, *S*_*i*_ is the size of food retailer *i* at time period *t*, *M*_*it*_ is the organic food sales per square meter of food retailer *i* at time period *t*, *T*_*it*_ is the total food sales per square meter of food retailer *i* at time period *t*, and *p*_*i*_ is the percentage of food sales per square meter that are organic of food retailer *i* in time period *t*.

### Explanatory (independent) variables

Based on the findings from the literature review and the theoretical background, we selected several explanatory variables to test the model. This includes data on the sociodemographic characteristics of the residents, voting behavior, and the surrounding physical environment (infrastructure). In some cases, further transformations were necessary (to be discussed in the following sections).

#### Sociodemographic characteristics

The following sociodemographic data describing Hamburg residents were gathered from *Statistik Nord* for the period 1999–2019: gender, age, and income. Data on the education and occupation levels of the residents were not available.

#### Voting behavior

Data on voting behavior were also gathered from *Statistik Nord* for the period 1999–2019. This describes the percentage of the population that voted for each party during national elections in Germany. The data include voting behavior for the five main political parties: *CDU*, *SPD*, “*Die Grünen”* (Greens), *FDP*, and *“Die Linke”* (Left Party). The Greens and Left Party have the strongest focus on climate politics and sustainability in their agendas [92].

The Greens are a mainstream left-center party in Germany built on an environmentalist platform. Their voter base is often categorized as urban, well-educated, or high-income earners [93]. The party has strongholds in larger cities in Western Germany, especially those with a large university presence [94]. The Left Party is considered the far-left party in Hamburg and Germany (of the political parties with parliament seats). They have increased in popularity in specific Hamburg areas over the last 20 years. The Left Party is considered by political scientists to be a “radical” party, with highly engaged voters in their beliefs and attitudes toward social and environmental issues [94].

Both the Greens and Left Party include sustainability and environmental topics related to food production as integral parts of their core platforms. This includes precise suggestions for climate-friendly agricultural redesign to increase the share of organic farming to 30% (Greens) and 25% (Left) by 2030 [92]. Both parties also called for subsidies in the farming sector to be tied to environmental criteria, such as the Green Deal or Paris targets [27]. A recent report from DIW Econ (2021), a climate economics consulting firm based in Berlin, analyzed the five main parties in Germany (CDU, SPD, FDP, Left Party, and Greens) based on different criteria across five sectors of climate politics (energy, industry, transport, building, and agriculture) and assigned points (0–4) based on the different parties’ commitment to environmental issues. In terms of support for organic agriculture, the Greens and Left Party both reached the highest score (4), followed by CDU (2), SPD (1), and FDP (0) [92]. We tested all parties and included voting for the Left Party and the Greens as variables in the model.

#### Physical neighborhood characteristics

Based on the literature review of accessibility to organic food, the following data on physical characteristics were included in the model: land use and public transportation stops. For public transportation, we counted public transport stops within one square kilometer of the shop. For land use data, we considered the type of land use (residential, commercial, mixed-use, and green/rural) where each shop was located.

## Model and methodology

Spatial dependence occurs when individuals are more (or less) like individuals closer to them than those farther apart and is rooted in Tobler’s First Law which states, “Everything is related to everything else, but closer things more so,” suggesting, according to Anselin and Bera (1998), that “spatial dependence to be the rule rather than the exception” [95]. In addition, a common assumption in statistical analysis is the use of a random sample or that variables are independent and identically distributed (i.i.d.), known as complete spatial randomness in a spatial sample. However, this is often violated in spatial datasets owing to spatial dependence, which can bias the estimation and inference. The inclusion of spatial data implies that standard linear modeling may result in measurement errors. Thus, we accounted for spatial autocorrelation through the model specification and estimation.

We developed a spatial pooled cross-sectional model to account for spatial dependence and identified target areas that may require more motivation to purchase organic foods. This is based on the SLM model with added complexity to account for space and time. The SLM (and more complex spatial models) includes observations of individual units (in our case, retail grocery stores) *i = 1…N*, pooled across several time periods *t = 1…T*. The steps of the analysis include (1) generating a spatial weight matrix, (2) testing for spatial effects, and (3) model specification.

### Step 1: Spatial weights matrix

We first define a spatial weight matrix, *W*, according to Anselin et al. (2008), based on a fixed distance (all features within the determined threshold distance impact each other) to express the interaction between location *i* and location *j* within each time period, *t*. We calculated the distance band to account for maximum spatial autocorrelation, which varies yearly with a mean of 3,644 meters, using the *Incremental Spatial Autocorrelation* tool in *ArcGIS Pro*, based on Global Moran’s *I* statistic for spatial autocorrelation. The spatial weight matrix layers for each year were combined to form a single matrix for further analysis. This is an *N* × *N* positive matrix for all shops in the study with a zero diagonal (no self-neighbors). The rows are standardized such that the given weight, *w*_*ij*_, of any row, is then divided by the row sum such that the sum of each row-standardized weight equals one.

**Fig 2:**
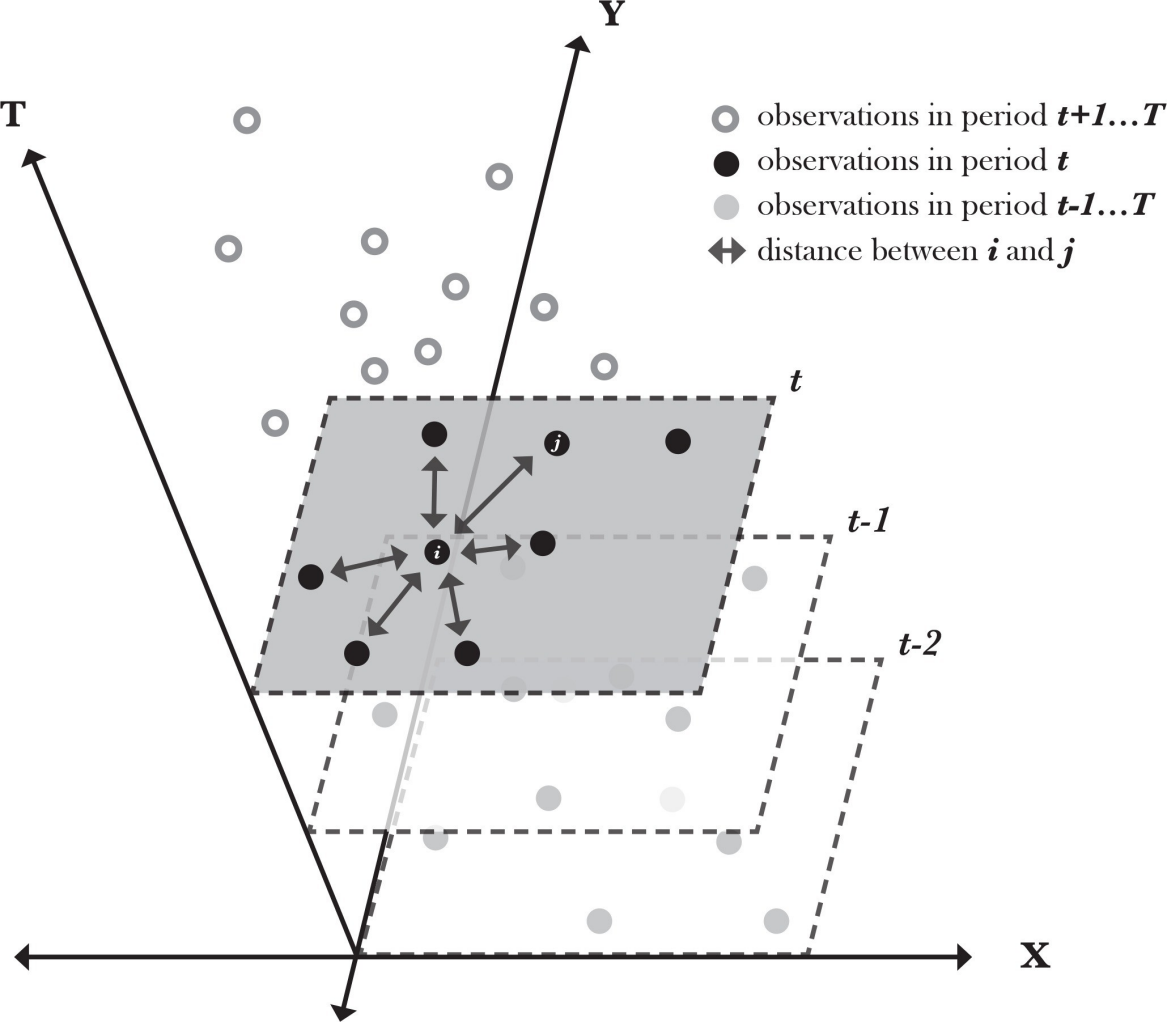
Schematic representation of the multidirectional spatial effect, *w*_*ij*_, for a given time period, *t*, and a particular point. Based on figure from study by Dubé and Legros [97].

The last step in refining the dataset is to remove any isolates (shops with no neighbors) that are not included the spatial weight matrix, according to Anselin and Bera (1998). When a shop has no neighbors within the distance band range, it is considered an isolate and removed from the dataset (n = 17). The spatial weight matrix layers were collected in a discrete-time structure (every two years for each time period, *t*) and compiled into one spatial weight matrix for the total sample *N x T*. The multidirectional spatial relationship between shop *I* and neighbors *j* for the given time period *t* is illustrated in Fig 2. This matrix is then used to construct a new variable that includes the weighted average of neighboring observations [96]. This is incorporated differently, depending on the model specification, as discussed in the following section.

### Step 2: Testing for spatial dependence

Next, we tested for spatial dependence using the Global Moran’s *I* test for the regression residuals and the Monte Carlo simulation of Moran *I* (with 1000 simulations). The results indicated spatial autocorrelation in the model, which was highly significant (p-values 4.034e-09 and 0.001, respectively). This indicated spatial dependence in the model residuals as well as potentially in the dependent and explanatory variables not yet confirmed. We ran a Lagrange multiplier (LM) test [99] for the error and lag terms to determine the most suitable spatial model for estimating our regression. The results indicated significant spatial dependence in the error term, as shown in Table 1. This also implies no spatial dependence in the dependent variable; thus, we can exclude this from the model specification.

**Table 1:** Results of the LM tests for the error (LMerr) and lag (Lmlag) terms.

Test name	Term	Diagnostic	p-value	
*Lmerr*	Error	32.281	1.334e-08	***
*Lmlag*	Lag	15991	0.206	

### Step 3: Model specification

We first estimated the data using an SLM as a reference, in which spatial effects are not accounted for.

**Putting this into formal notation:**

y=Xβ+ε, (3)

where *y* is an *N x 1* vector of observations of the dependent (explained) variable, *X is* the *N x K* matrix of observations of the independent (explanatory) variables, *β* is the *K* × 1 vector of undetermined coefficients, and *ε* is the *N x 1* vector of the error terms.

The results of the LM imply that we need to account for spatial effects in the error term using the spatial error model (SEM), in addition to testing spatial effects on the independent variables with the spatially lagged X model (SLX), which is not included in the LM tests. These models are also used to develop a more complex spatial Durbin error model (SDEM) that incorporates the lag of the independent variables (SLX) into the SEM model. As no spatial effects were identified in the dependent variable (lag), the spatial lag model (SAR) or the more complex nested spatial Durbin model (SDM) were not included.

The SEM, also referred to as the spatial autoregressive error model, incorporates spatial dependence in the disturbance (error) terms and is formulated as follows:

y=Xβ+u, (4)

u=λWu+ε (5)

where *λ* (lambda) is the spatial autoregressive coefficient for the error lag *Wu*, and *ε* is the uncorrelated and homoscedastic error term.

SLX considers the spatial spillover effect of neighbors on the independent variables and is expressed as follows:

y=Xβ+WXθ+ε (6)

where *X* is the *N* × *K* matrix of explanatory variables associated with the *K* × 1 parameter vector *β*, which measures the direct effects of the *X* variables, *WX* is the matrix of exogenous spatial lags, and *θ* is the *K x 1* vector of parameters which determine the marginal (indirect) effects of the independent variables from neighboring observations.

Similarly, the SDEM incorporates the lag of the independent variables (Eq 6) into the SEM model, expressed as

y=Xβ+WX+u, (7)

u=Wu+ε (8)

**Fig 3:**
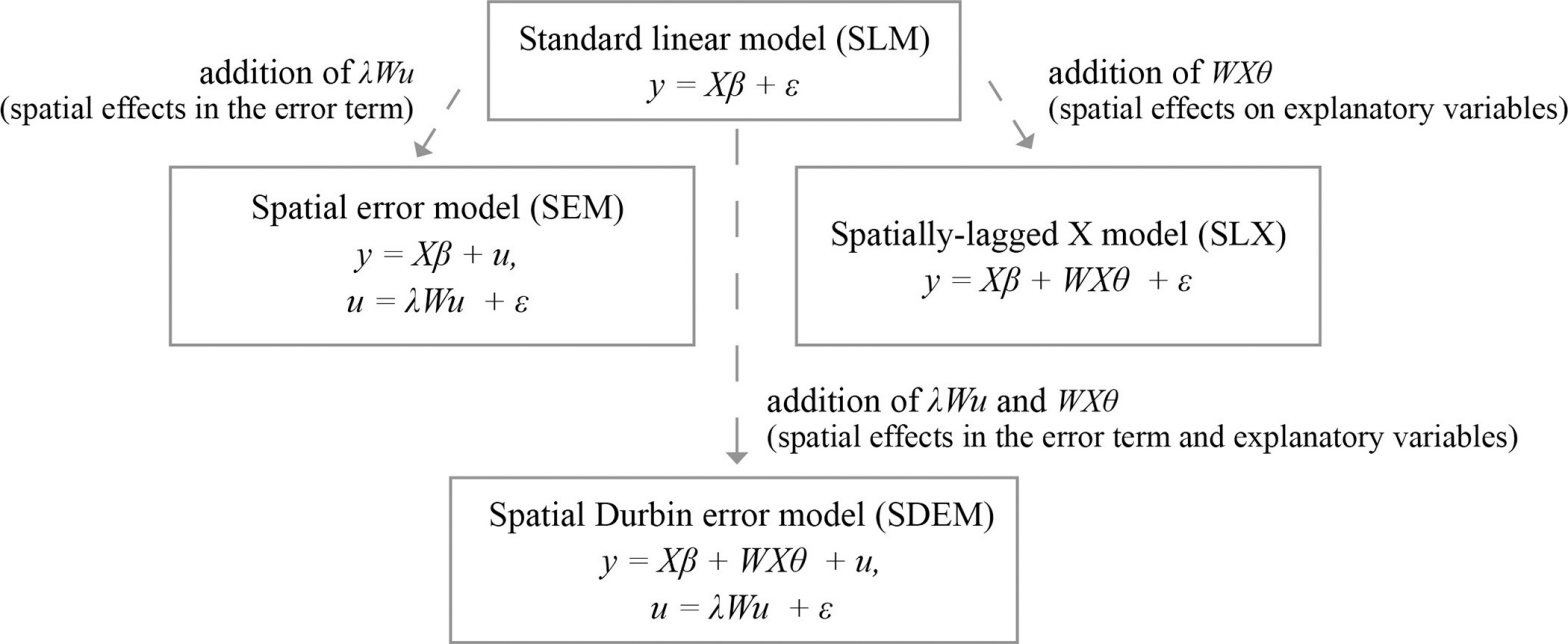
Relationship between SLM and more complex SEM, SLX, and SDEM models, based on figure from study by Golgher and Voss [98].

The relationships among SLM, SEM, SLX, and SDEM are shown in Fig 3.

#### Data cleanup and transformations

We tested for multicollinearity between all potential explanatory variables using the Pearson’s correlation test. If variables were more than .40 correlated with another (negative or positive), indicating a “moderate correlation” or stronger, then they were disqualified from being in the model together, and one was selected based on model performance according to Schober and Schwarte (2018). The data included observations of the entire city-state of Hamburg (both at the street and district levels, depending on the source) across the entire time period (1999–2019). When no data was available from industry or company reports for that specific year, we imputed the missing data (less than 15% of the total dataset of explanatory variables) based on the values of the previous or later year(s). This was relevant to two variables: income and voting. Owing to privacy laws, data on income were not collected for the following years in our model: 2015, 2017, and 2019. In addition, since voting took place every four years, it was impossible to gather data for each year of the study; thus, the missing data were imputed based on the trend from all voting between 1999–2019.

**Fig 4:**
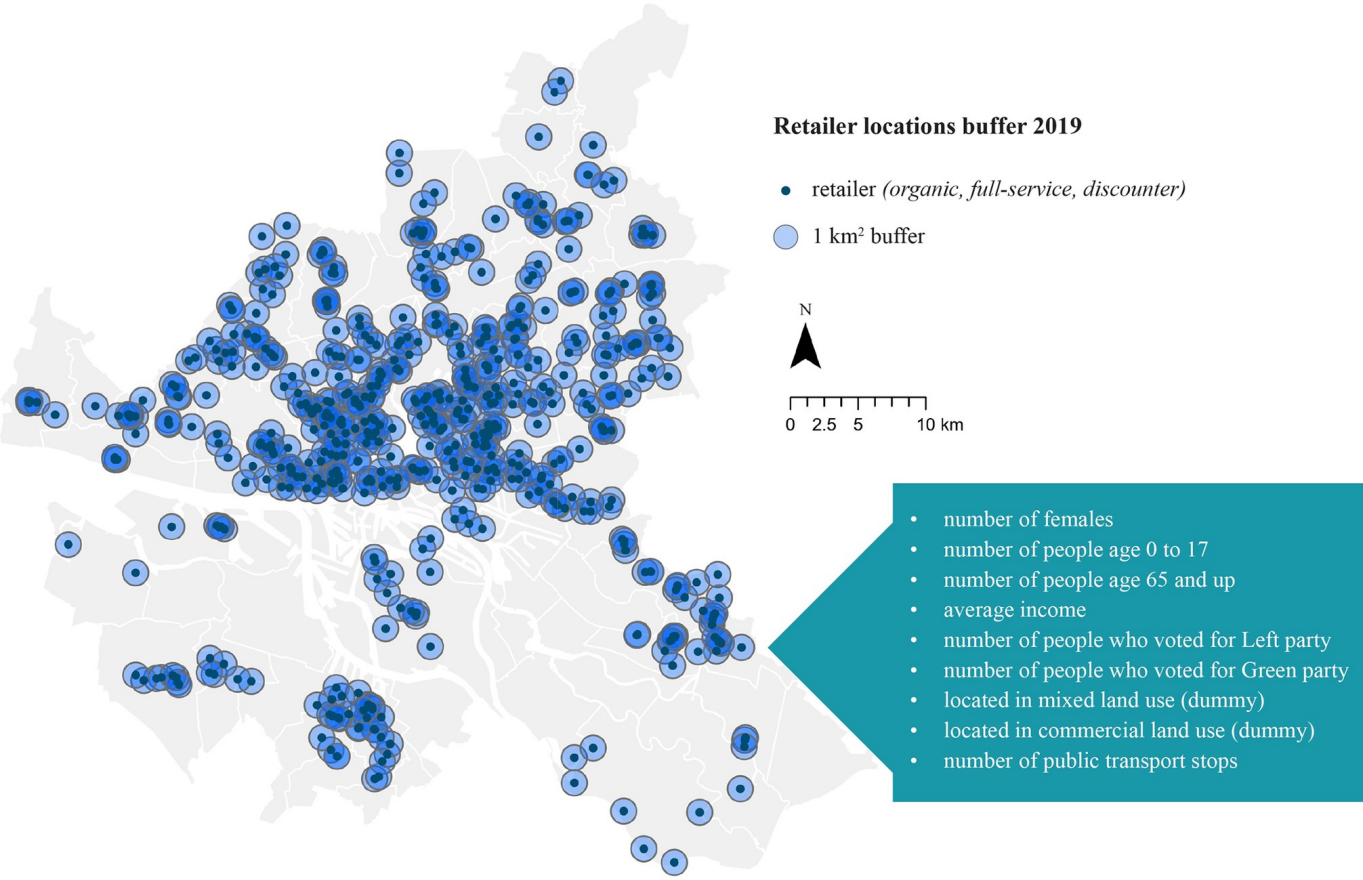
Buffer area surrounding each retailer location in 2019 (Hamburg, DE), with description of data (independent variables), included within each circle. Source: Maps generated with own data in ArcGIS Pro.

The data for sociodemographic characteristics and voting behavior were also gathered at the more aggregated neighborhood level (with one observation for each of the 99 districts per year over 20 years); thus, further steps were needed to make it applicable to the shop level. One possibility was to give each shop the characteristics of the neighborhood in which it was located. However, this does not account for the influence of neighboring areas, particularly if shops are located on or near the border. Therefore, we generated a one-square-kilometer buffer around each shop and took the mean of all sociodemographic and voting data within this buffer, as well as the mean population density (number of people per square kilometer). As the sociodemographic and voting data were expressed as a percentage (of the total population), we multiplied this by the mean population density of the buffer area to convert the percentages to integers (representing an individual) to create more comparable results. However, the population of some areas was significantly higher than others; thus, comparing values as a percentage of the population could be misleading. The result was then expressed as the number of people corresponding to the different variables (e.g., the number of females) within one square kilometer of each shop. The only exception was income, which was expressed as the average income per person. This is illustrated in Fig 4.

#### Further explanatory variables

In addition to sociodemographic, voting, and physical neighborhood data, we included other explanatory variables to improve the model. The first was time, a classical trend variable in which we assigned a dummy variable for each period in the study (i.e., 1 = 1999, 2 = 2001, …, 11 = 2019) to help explain and illustrate the change in organic food sales over time. In addition, we included two variables to show the interaction between the shop size and the percentage of organic food sales compared to the total food sales of a specific shop. The purpose of these variables was to help the model explain the high difference in organic food sales between organic food retailers (in which 100% of sales are organic) and conventional retailers (in which the organic food sales range from a minimum of 1% in 1999 to a maximum of 5% in 2019), which will be discussed in more detail in the next section. The descriptions of the explanatory variables included in the model are presented in Table 2. All variables were continuous, and the values for gender, age, voting behavior, and public transport stops are the sum of all people (i.e., number of females, number of people voting for each political party, based on the transformations previously discussed) or stops (public transport stops) within one square kilometer of the shop itself, whereas the income was an average.

**Table 2:** Description of the model’s explanatory variables. For the variables female, age0to17, age65Up, income, votinglinke, votinggruene, and hvv this corresponds to the total number of units within one square kilometer of each shop.

Variable name	Description
*time*	Time period of the observation
*size*	The size (in m^2^) of each shop
*perorg*	The percent of total food sales that is organic
*female*	Number of residents that are female
*age0to17*	Number of residents aged 0 to 17
*age65up*	Number of residents over age 65
*log10(income)*	Average income of residents
*votinglinke*	Number of residents that voted for the Left Party
*votinggruene*	Number of residents that voted for the Greens
*lumixed*	Dummy variable for land use type mixed (residential and commercial)
*lucommercial*	Dummy variable for land use type commercial
*hvvcount*	Number of public transport stops

### Exploratory data analysis and treatment

First, we examined the distribution of organic food sales as the dependent variable. As organic retailers are allotted 100% of total sales for organic food products, the total organic sales from these retailers will be much higher than from conventional retailers, as discussed in the previous section. Furthermore, our sample included significantly more conventional retailers (n = 4722) than organic ones (n = 212). Thus, the frequency of (generally) comparatively lower values from conventional retailers will be more than 15 times higher than that of organic retailers. The abnormal distribution was apparent when we examined organic food sales summary statistics without transformation. To generate a normal distribution, we took the log10 of the total organic food sales of each retailer. The summary statistics of the dependent variables before and after the log10 transformation for the final dataset are presented in Table 3. Next, we tested for the normality of the explanatory variables and applied a transformation where necessary (we took the log10 of income, but the other variables remained as is).

**Table 3:** Summary statistics of the dependent variable (organic sales) before and after log10 transformation.

	Unit	Min.	1^st^ Qu.	Median	Mean	3^rd^ Qu.	Max.
*organic sales*	€	6,572	46,250	86,694	156,018	155,722	506,430
*log10 of organic sales*	€	3.82	4.67	4.94	4.95	5.19	6.71

Following the selection of the explanatory variables to be used in the model and the application of the log10 transformation, the Normal Q-Q plot (to test for normality) and scatterplot (to test for homoscedasticity) indicated that some outlying observations negatively influenced the performance of the model. We applied Cook’s Distance, identified influential outliers in the explanatory variables, and removed observations that were more than three times the mean (n = 284, approximately 5% of the total observations). Initial tests on the model indicated heteroskedasticity in the residuals (a common problem in cross-sectional datasets over time, especially with multilevel observations). However, once Cook’s Distance transformation was applied, the scatterplot indicated that the model could assume homoskedasticity.

## Estimation results and analysis of spatial effects

We first estimated each of the above-mentioned models and then compared the results. This included testing direct and marginal effects (described in the following section). Next, we selected the best model based on the study’s overall objective (which model would best address the influence of the surrounding area of each shop), as well as a goodness-of-fit test. All models had a direct effect on the explanatory variables. The main findings indicated that over the 20 years, the number of older people (aged 65 and above), higher average income, and increased voters for the Green and Left parties were positively correlated with more organic food sales. Regarding accessibility and infrastructure, shops located in a mixed land use and commercial area and those with more public transportation stops nearby were positively correlated. However, the number of females and children (aged 0–17) living near the shop was negatively correlated with organic food sales.

### Testing and comparing model specifications

The estimation results for the SLM, SEM, SLX, and SDEM are presented in Table 4. The estimate of the direct effect of the explanatory variables is listed in the first section of the table for SLM and each spatial model, as well as the spatial autocorrelation parameter for the error terms (*λ*). This parameter indicates that when the average of our neighbor’s residuals is higher by one unit, our residuals will be higher by *λ*, and are included in the models that account for spatial effects in the error term (SEM and SDEM). The estimate for the indirect effect (indicated with a “lag.” before the variable name) is presented in the second half of Table 4 representing local spillover to neighboring observations for the explanatory variables (SLX and SDEM). All four models are presented first as a robustness check but also to compare the results of the models, especially the SLM, with the spatial models.

**Table 4:** Summary results and statistics for estimation of SLM and spatial models (SEM, SLX, SDEM).

Variables	SLM	p-value		SEM	p-value		SLX	p-value		SDEM	p-value	
*intercept*	4.04E+00	2.00E-16	***	4.03E+00	2.20E-16	***	4.02E+00	2.00E-16	***	4.02E+00	2.20E-16	***
*time*	5.32E-02	2.00E-16	***	5.33E-02	2.20E-16	***	5.34E-02	2.00E-16	***	5.34E-02	2.20E-16	***
*size*	5.39E-04	2.00E-16	***	5.38E-04	2.20E-16	***	5.37E-04	2.00E-16	***	5.37E-04	2.20E-16	***
*perorg*	1.31E+00	2.00E-16	***	1.31E+00	2.20E-16	***	1.31E+00	2.00E-16	***	1.31E+00	2.20E-16	***
*female*	-2.23E-05	0.0228	*	-1.99E-05	0.041674	*	-1.98E-05	0.043626	*	-2.00E-05	0.0401585	*
*age0to17*	-1.49E-05	0.0024	**	-1.54E-05	0.001599	**	-1.62E-05	0.001003	**	-1.63E-05	0.0008825	***
*age65up*	4.55E-05	2.00E-16	***	4.53E-05	2.20E-16	***	4.53E-05	2.00E-16	***	4.53E-05	2.20E-16	***
*income*	1.08E-06	8.23E-11	***	1.03E-06	5.24E-10	***	1.04E-06	6.69E-10	***	1.05E-06	3.82E-10	***
*votinglinke*	4.39E-05	2.00E-16	***	4.39E-05	2.20E-16	***	4.40E-05	2.00E-16	***	4.40E-05	2.20E-16	***
*votinggruene*	4.29E-06	0.0191	*	4.58E-06	0.011945	*	4.42E-06	0.015977	*	4.39E-06	0.0160681	*
*lumixed*	7.87E-03	0.034	*	7.58E-03	0.041305	*	7.35E-03	0.049597	*	7.35E-03	0.0481156	*
*lucommercial*	1.53E-02	0.0319	*	1.61E-02	0.024409	*	1.78E-02	0.01333	*	1.78E-02	0.0129879	*
*hvvcount*	1.02E-02	4.49E-05	***	1.06E-02	2.55E-05	***	1.03E-02	4.32E-05	***	1.03E-02	3.73E-05	***
*λ*				0.2553						0.2209		
*lag*.*intercept*							8.91E-01	0.009361	**	9.35E-01	0.0174578	*
*lag*.*time*							-5.46E-03	0.375572		-3.90E-03	0.5712644	
*lag*.*size*							2.40E-05	0.285432		1.82E-05	0.4739029	
*lag*.*perorg*							4.45E-02	0.304016		4.01E-02	0.4240525	
*lag*.*female*							-1.42E-04	0.008313	*	-1.46E-04	0.0175083	*
*lag*.*age0to17*							-1.13E-07	0.996137		-5.22E-06	0.8452659	
*lag*.*age65up*							1.71E-05	0.514982		1.61E-05	0.5888072	
*lag*.*income*							2.68E-06	0.000942	***	2.69E-06	0.0035274	**
*lag*.*votinglinke*							-5.51E-08	0.998385		-3.40E-06	0.9109482	
*lag*.*votinggruene*							-2.27E-05	0.034047	*	-2.32E-05	0.0543004	
*lag*.*lumixed*							5.91E-03	0.738698		9.74E-03	0.6270961	
*lag*.*lucommercial*							-2.86E-03	0.938082		3.62E-03	0.9307746	
*lag*.*hvvcount*							-1.74E-02	0.169639		-2.40E-02	0.0984227	
Statistics												
Observations	4934			4934			4934			4934		
R^2^ / Psuedo R^2^	0.919			0.919			0.919			0.920		
Log Likelihood				3496.79						3508.502		
Sigma 2				0.014						0.014		
Residual Standard Error	0.120						0.119					
F Statistic	4633.525						2233.251					
Wald Test				24.301						17.310		
LR Test				24.503						17.295		

### Model selection

The autoregressive coefficient (*λ*) was 0.25 and 0.22 in the SEM and SDEM models, respectively. This indicates spatial dependence in the model (and thus, we used a spatial model). Additionally, the spatial correlation in the error term also implies that there are unexplained clusters of variables not included in the model, which follows logic as there are some variables that were identified as drivers for increased organic food purchases (such as education or occupation level) that were not included because these data were not available. Therefore, we selected a model that accounted for spatial dependence in the error term (i.e., ruled out the pure SLX model). However, as the objective of the study was to identify the drivers of demand for organic food sales surrounding each shop, we also want to consider the potential spatial dependence of the explanatory variables on neighboring observations. Based on this, the SDEM was the best model, as it accounted for spatial effects in both the explanatory variables and error terms.

Subsequently, we compared the goodness of fit for all models using the likelihood-ratio test (LRT) for spatial linear models to determine whether adding complexity to the model (i.e., more parameters) makes the model more accurate. The LRT compares two hierarchically nested models, in which the null hypothesis (H0) states that we should restrict the model to a simpler model, and the alternative hypothesis (H1) states that you should not restrict but rather use the more complex model. After testing all models against each other, the results of the LRT indicated that we should reject the null hypothesis and use the more complex model, SDEM.

### Sensitivity to change

The estimation results indicate that several factors significantly influence the demand for organic sales, including spatial effects. However, the interpretation of the results is complicated because the variables were unstandardized, and we applied a log10 transformation to the dependent variable. Furthermore, although the variables were significant, this did not indicate the strength of the impact. Therefore, we report the sensitivity to change as a measure of the dependent variable’s sensitivity relative to a change in the independent variables. Some variables fluctuated considerably between neighborhoods (e.g., income or voting for the Left Party); thus, we first calculated the change in the dependent variable for the minimum and maximum observations of the independent variables. This can be interpreted as, “When the value of the variable is at its min/max, organic sales increase/decrease by *X*% compared to the mean (keeping all other variables constant at the mean).” We applied the same rationale to test the change in the dependent variable when the independent variables increased by 1% from the mean. The results are presented in Table 5.

**Table 5:** Change in organic food sales (in € and %) corresponding to the maximum and minimum values of each variable, and a 1% increase in the mean of each variable (when all other variables remain at the mean) for the SDEM.

	Statistics			Min		Max		1% Increase Mean	
Variables	min	mean	max	%change	euro(€)	%change	euro(€)	%change	euro(€)
*female*	5149	6695	7139	4.07%	6348	-1.11%	-1738	-0.17%	-265
*age0to17*	1038	2020	3764	2.06%	3216	-3.48%	-5427	-0.04%	-65
*age65up*	844	2407	4413	-8.29%	-12933	12.82%	20003	0.14%	216
*income*	11529	35097	140258	-3.04%	-4740	15.87%	24759	0.05%	73
*votinglinke*	130	1192	4478	-5.62%	-8764	21.73%	33895	0.07%	104
*votinggruene*	236	2061	11759	-1.01%	-1572	5.68%	8854	0.01%	18
*hvvcount*	0	0.58	6	-0.76%	-1183	7.59%	11834	0.01%	12

To calculate the value of sales when all variables are at the mean, we define A as:

A:=10^(Intercept+esttime×meantime+estsize×meansize+estperorg×meanperorg+estfemale×meanfemale+estage0to17×meanage0to17+estage65up×meanage65up+estincome×meanincome+estvotinglinke×meanvotinglinke+estvotinggruene×meanvotinggruene+estlumixed×meanlumixed+estlucommercial×meanlucommercial+esthvvcount×meanhvvcount) (9)

Further steps are required to calculate the sensitivity (change in sales) for the minimum, maximum, and 1% mean increase, and each variable is calculated using a different equation. Using voting for the Left Party (*votinglinke*) as an example, the equation for changes in sales is

**When voting for the Left Party is at a minimum**:

$$C_{sales,minvotinglinke}=(A–({est}_{votinglinke}\times {mean}_{votinglinke})+{mean}_{votinglinke}\times {min}_{votinglinke})–A$$ (10)

**When voting for the Left Party is at a maximum**:

$$C_{sales,maxvotinglinke}=(A–({est}_{votinglinke}\times {mean}_{votinglinke})+{mean}_{votinglinke}\times {max}_{votinglinke})–A$$ (11)

When voting for the Left Party increases by 1%:

$$C_{sales,1\%votinglinke}=(A-({est}_{votinglinke}\times {mean}_{votinglinke})+{mean}_{votinglinke}\times 1.01)-A$$ (12)

The strongest effect occurred when voting for the Left Party (*votinglinke*, interpreted as the number of people voting for the Left Party surrounding the particular location) was at a maximum. According to Table 5, organic food sales are nearly 22% higher in areas with the most support for the Left Party compared to the mean. The change in sales of the maximum value for voting for the Greens Party *(votinggruene)* was not high (5.68%) but was still notable. The results of the sociodemographic variables, such as older people (*age65up*) and income (*income*), also indicated a positive effect. When average income was at the maximum observation value, organic sales increased by approximately 13% and 16% for age 65 and above and income, respectively. The results also indicated that having more children (*age0to17*) or women (*females*) in the surrounding area had a negative effect, although this was small. For minimum values, the strongest effect occurred when fewer older people lived nearby (-8.29%), followed by left-party votes (-5.62%).

Finally, an increase in public transportation stops (*hvvcount)* apparently had both a significant result and an effect for areas with the maximum number of stops. Therefore, increased accessibility to shops may correlate with increased organic food sales. However, the current mean number of stops located within one kilometer of shops was 0.58, whereas the maximum was six. This implies that some areas have very high accessibility compared to the mean, but it is also realistic to assume that new public transportation stops could be installed in underserved areas, which could increase sales. We also showed changes in sales with one, two, and three additional train stop(s). This is presented in Table 6. The results indicate that by adding more stops around a shop, organic sales could increase by 1.33% (one stop), 2.69% (two stops), or up to 4% (three more stops). The formulas are as follows for a one-stop increase (with a two-stop increase calculated by “+ 2” and three stop increase calculated by “+ 3”:

$$C_{sales,+1hvvcount}=(A-({est}_{hvvcount}\times {mean}_{hvvcount})+{mean}_{hvvcount}+1)-A$$ (13)

**Table 6:** Change in organic food sales (in € and %) corresponding to a one, two, and three-stop increase for the variable hvvcount (when all other variables remain at the mean) for the SDEM.

	Statistics	1 Stop Increase		2 Stop Increase		3 Stop Increase	
Variables	Mean	%change	euro(€)	%change	euro(€)	%change	euro(€)
*hvvcount*	0.58	1.33%	2071	2.69	4192	4.08%	6364

## Discussion

This study aimed to analyze the drivers of organic food sales, including identifying groups that positively correlate with increased organic food sales over time. In contrast to the current research, we introduced a new methodology to achieve this objective and used spatial data (to account for spatial effects and identify target areas) collected over 20 years (to model development over a longer period). By considering a macro approach, we improved the robustness of results and removed bias associated with surveys or self-reported attitudes or behavior, which has been the basis of most studies to date. Instead of grouping consumers according to their stated attitudes or behavioral intentions towards organic food or sustainability, we identified the groups that had a positive relationship with organic food sales over time within an entire region based on the data.

### Interpretation of results and recommendations

The results of the study also reconfirm the findings of the literature review in terms of attitude (expressed as voting for a particular party on a climate politics platform), namely, that attitude plays a role in the demand for organic food. We found that demand for sustainable food products is driven predominantly by attitude-related factors, which are most closely correlated to more “convinced” consumers who exhibit positive attitudes towards sustainable and environmental topics. This is evidenced by the significant positive correlation between organic food sales and voting for the Green Party and Left Party. However, the correlation and sensitivity to change of the Left Party were stronger (organic food sales were 22% higher than the mean in areas where voting for the Left Party was highest, compared to the Greens at 6%). This may seem counterintuitive, as the Greens voters tended to have a higher income and the strongest climate politics compared with all the other parties (although the Left Party also strongly focused on climate politics, as discussed in previous sections). One explanation for this, which further corroborates with the literature, is that the Left Party is considered more “radical,” where supporters are very convinced of their attitudes (pro-environment) and behavior thus follows. In contrast, the Greens are a more mainstream party, in which supporters may indicate a preference for sustainability and climate politics but are not as convinced and thus have insufficient WTP for organic products. This provides potential evidence for the attitude–behavior gap without using self-reported data.

These results also help enrich the current scientific literature. Unlike many sociodemographic factors that tend to remain constant (or change slightly) over a person’s lifetime, attitudes can be shifted over time with exposure to new knowledge or experiences, as evidenced by Papp (2022). Regarding sociodemographic characteristics, the results mirror those of the literature review in that they were mixed. The only sociodemographic characteristic consistently associated with a preference for organic food or sustainability in the literature is education level, which was not available in our database. Our study indicates that factors such as accessibility, education (in the error term based on the literature), gender, age, and income also play a role.

**Fig 5:**
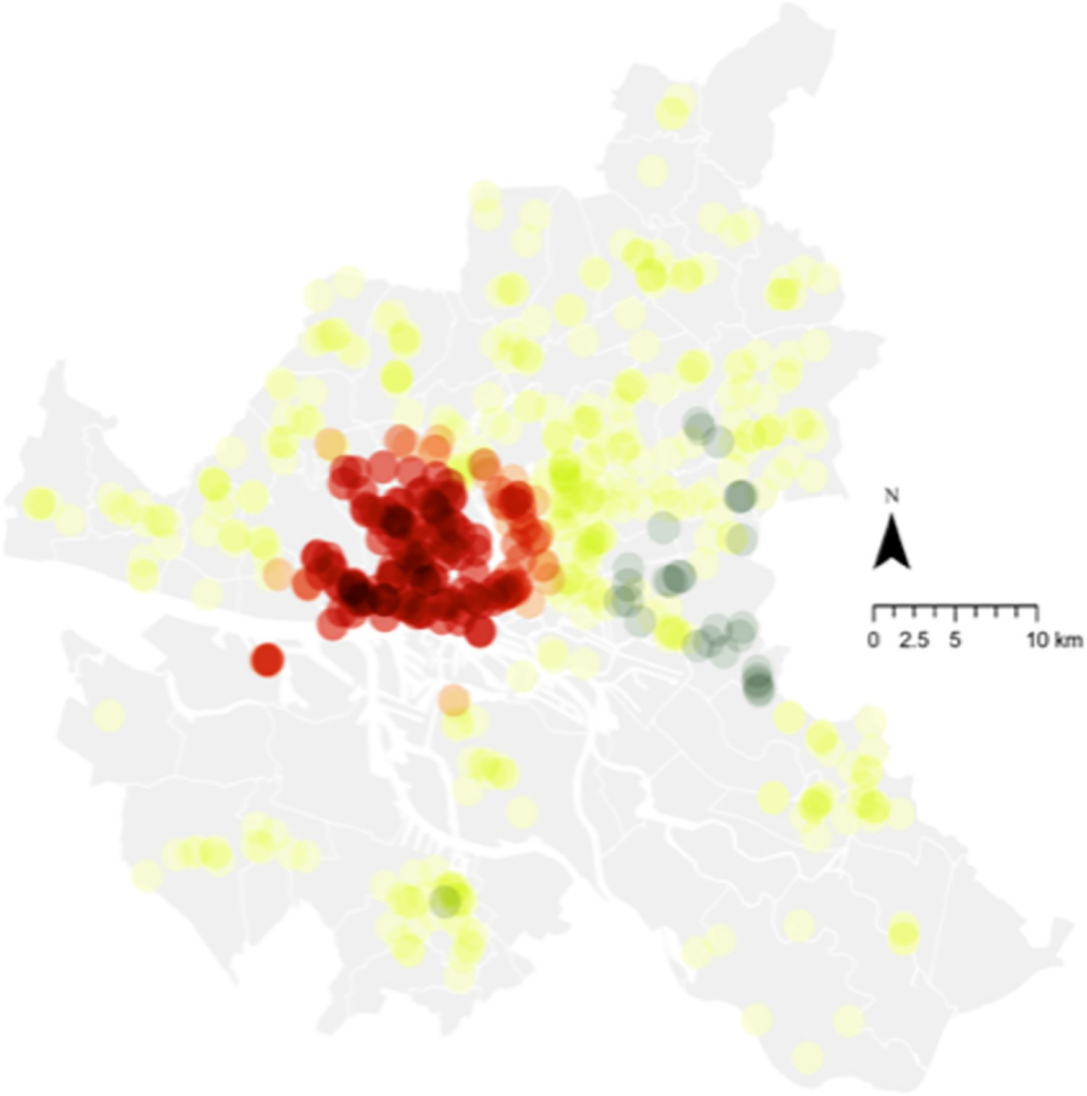
Hotspot analysis of organic food sales (2019). Source: Maps generated with own data in ArcGIS Pro.

**Fig 6:**
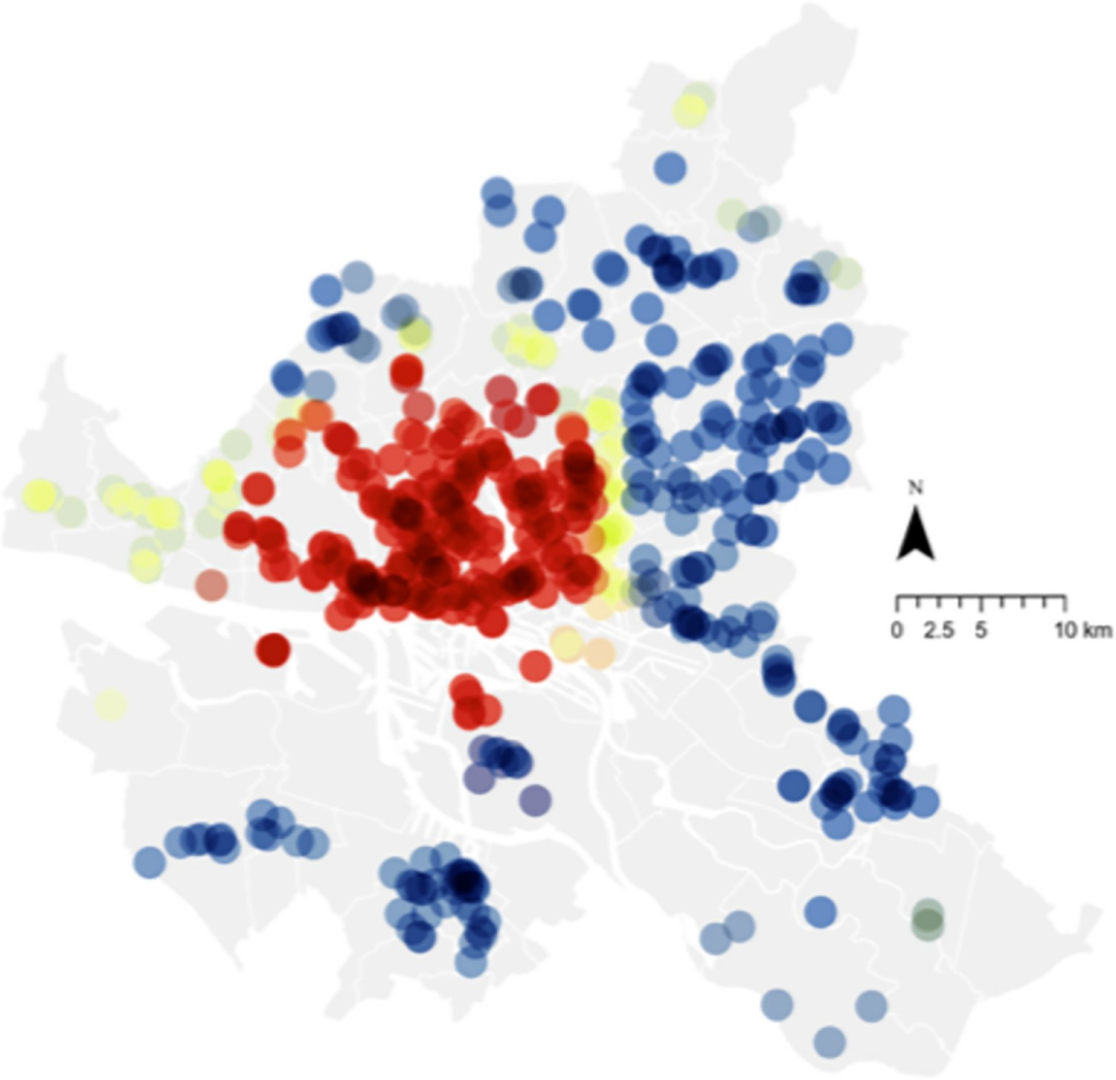
Hotspot analysis of voting for the Left Party and the Green Party (2019). Source: Maps generated with own data in ArcGIS Pro.

**Fig 7:**
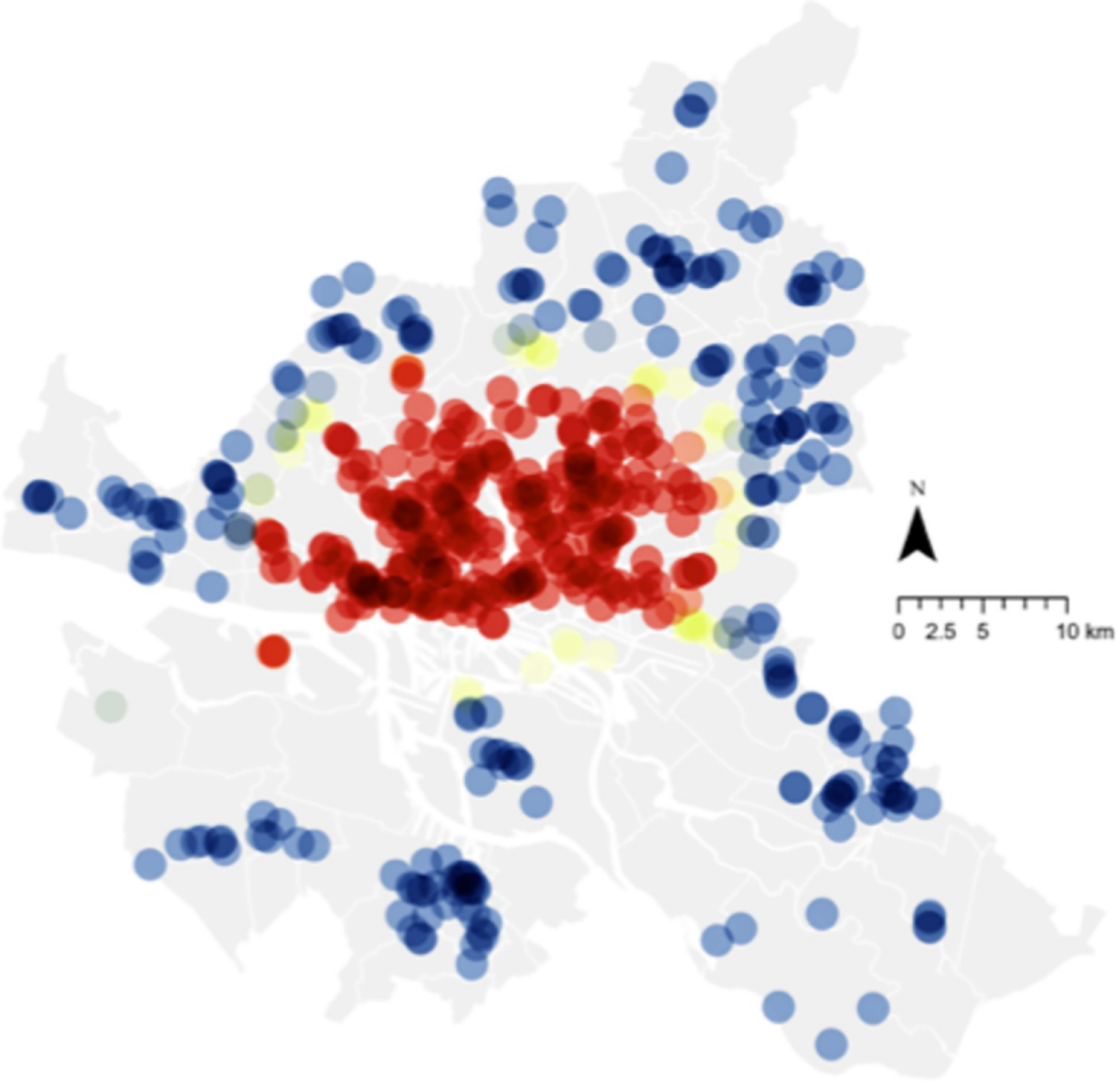
Hotspot analysis of population density (2019). Source: Maps generated with own data in ArcGIS Pro.

**Fig 8:**
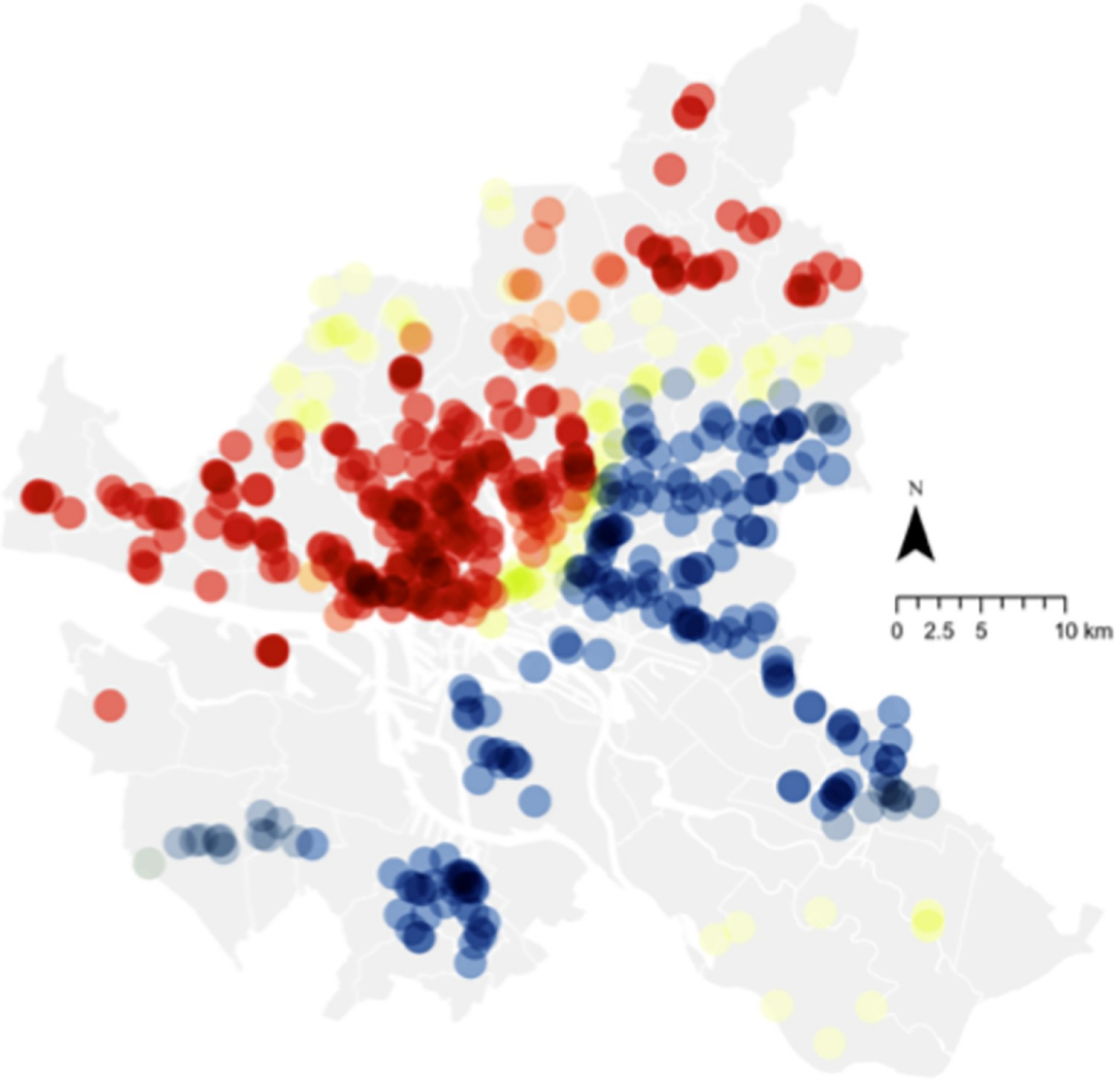
Hotspot analysis of income (2019). Source: Maps generated with own data in ArcGIS Pro.

**Fig 9:**
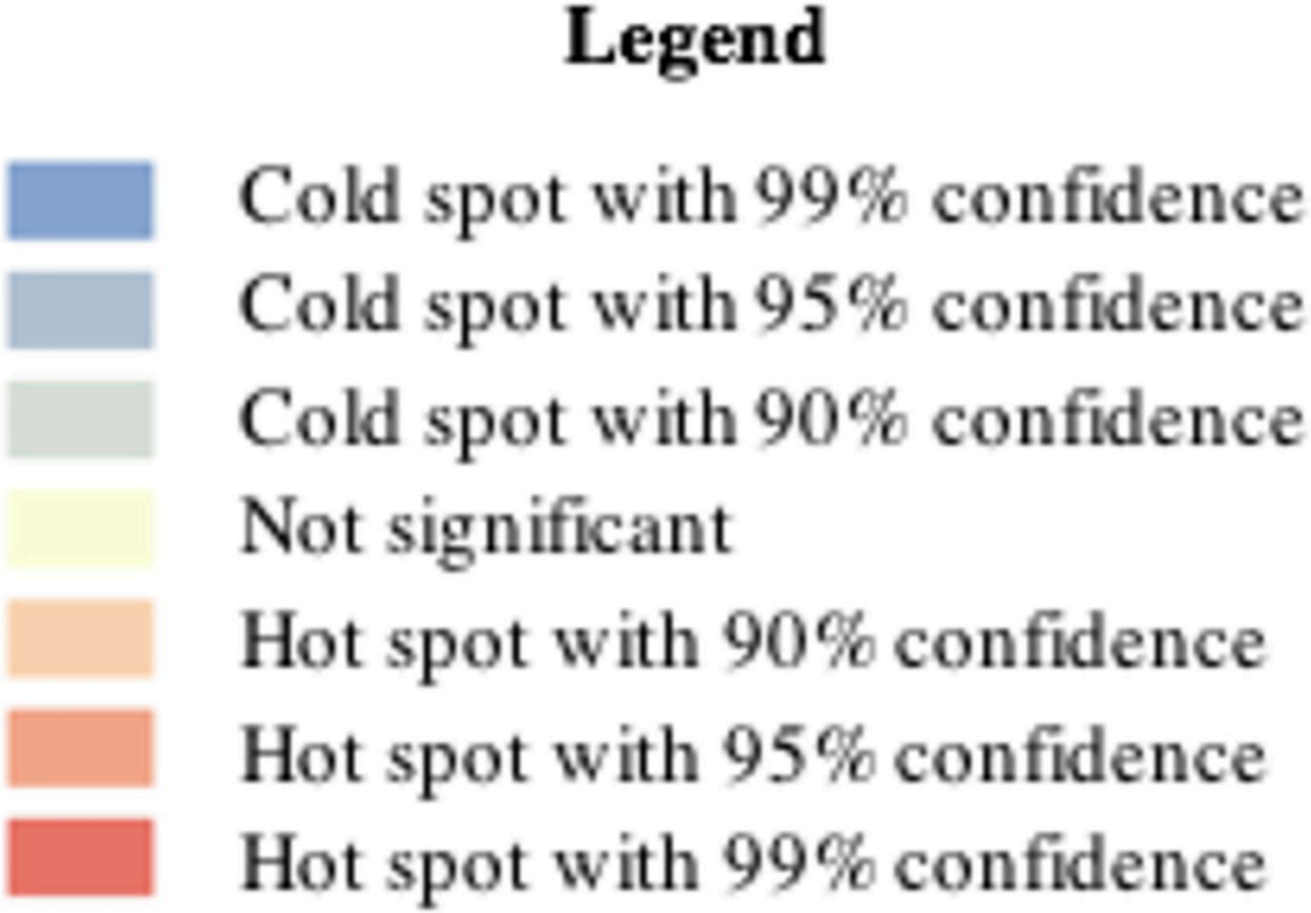
Legend for Figs 5–8.

An example of the spatial relationship between voting behavior and organic food sales for 2019 is illustrated in Figs 5 and 6. Red spots indicate highly significant hotspots, whereas blue indicates highly significant cold spots. Fig 5 shows the hotspot for organic food sales in Hamburg in 2019, concentrated in the southwest corner of the city above the border with the *Elbe River*. This is closely illustrated by the hotspots of voting for the Left or Green party for the same year, which provides a further visual illustration of the relationship (albeit for a single year and thus not representative of the model). Comparing these two figures to Fig 7, which illustrates the hotspot of population density, it is apparent that while the central area of Hamburg is highly dense, organic food sales are concentrated only on the western side. This is similar to voting, although the voting hotspot was larger. This becomes more interesting when income is considered, as illustrated in Fig 8. While the hotspots for population density are dispersed throughout the center of the city, hotspots for income are only on the western side, as with organic food sales. This may imply the moderating role of income on attitude and organic food sales, which is not directly dealt with in this study but is a notable point for further research. Please see Fig 9 for legend.

## Theoretical and practical implications, study limitations, and conclusions

The current literature indicates that self-reported behavior is not always transferred to true purchasing behavior (i.e., the attitude-behavior gap). Thus, several studies have combined true purchase data and self-reported preferences for sustainability and organic foods. However, these studies still rely on surveys in which consumers may overestimate preferences for organic/sustainable foods; therefore, studies need to describe organic consumers that have not been collected via surveys (i.e., no self-reported data). By considering this macro perspective to gather data and model relationships, we identified the drivers of organic food sales without using self-reported data, a key challenge identified by current research [42, 44]. Furthermore, by pooling the data over 20 years, we identified robust drivers and avoided the potential bias of data collected at a single point in time or over a few years. Additionally, by developing a spatial model, we surpassed the standard SLM and accounted for unobserved spatial effects (e.g., spatial dependence) of neighboring areas on the dependent variable, explanatory variables, and the error term that had not yet been addressed in the current research. The results also further contribute to research on the attitude-behavior gap [41], as our study indicates that positive attitudes towards environmental sustainability have a significant relationship with increased organic food sales. However, the actual effect of this is highly dependent on how “convinced” consumers are. Finally, we expanded the literature on voting behavior and ecological preferences by linking preferences for political parties and increased organic food sales.

In terms of practical implications, this analysis can support relevant stakeholders, such as companies and governments, who want to increase their share of organic food sales. For companies such as food retailers or those operating in the food supply chain, the results spatially illustrate target areas for new shops (based on where sales currently thrive or where there may be a lack of shops) to increase sales and profits. In addition, we illustrated the need for “convincing” consumers. This may be achieved through increased knowledge sharing at the shops or as part of a city-wide initiative. City planners and policymakers may use spatial data to identify regions needing further motivation and tailoring campaigns to local residents. The maps in Figs 5–8, for example, illustrate the potential to derive insights from the spatial data itself, as well as to identify potential areas for future research.

However, this study has some limitations. The first is the availability of data. To consider a macro overview, some of the data had to be reasonably inferred based on relevant sources (organic food sales) or imputed (<15% of data from the independent variables). Furthermore, although the macro-overview provides unique advantages, it also complicates the model. There could be specific characteristics of neighborhoods that are not captured by the model and result in biased observations, such as data on education levels. Although the study deals with data for an entire population over an extended period, it was gathered in Germany, which may not be generalizable to other areas. However, the results of the study by Nawrotzki (2012) did find that the role of political ideology in supporting environmental issues is comparable in countries with similar characteristics. This could imply that the results are generalizable to other highly developed capitalist nations with good environmental conditions.

Another limitation is the scale of the data (common in spatial statistics). The data used have a spatial component and thus require some transformations to come to a common scale. For example, data for sociodemographics and voting behavior were at the district scale, whereas data for physical neighborhood characteristics and organic food sales were at the street level. To address this, we disaggregated sociodemographic and voting behavior by creating an average value within a one-kilometer radius of each shop based on the average population density, thus accounting for values from potentially neighboring districts. However, this represents an average value surrounding each shop, which may not be the reality at a specific street level.

In conclusion, shifting the food system to one that is more sustainable requires changes in the demand and supply sides, as well as bottom-up and top-down approaches. Understanding the factors that positively correlate with increased organic food sales over time for an entire population can help guide policymakers, industry, or educational organizations to further increase this transition. In this study, we analyzed the relationship between organic food sales and the area surrounding the shop, that is, measures for the sociodemographic characteristics and attitudes of residents, as well as the physical neighborhood surrounding the shop. The results indicated that attitude towards sustainability strongly correlates with the demand for organic food, but WTP (being “convinced”) is also a key element. This was enriched by spatial data, which accounted for spatial effects as well as allowed targeted interventions to increase the consumption of sustainably produced goods, especially in areas that need further motivation or knowledge. Understanding how to engage consumers in increasing the demand for sustainable food products is a key next step forward and an opportunity for further research.
